# GP Trainees experience of learning opportunities and support mechanisms on the GP training programme: a qualitative study

**DOI:** 10.15694/mep.2020.000270.1

**Published:** 2020-12-02

**Authors:** Nadia Fisher-Plum, Catherine Woods, Johnny Lyon-Maris, Sally Curtis, Geraldine Leydon, Hazel Everitt

**Affiliations:** 1 University of Southampton; 2 Health Education England Wessex

**Keywords:** GP Trainees, Support, Learning Opportunities, Training, General Practice

## Abstract

This article was migrated. The article was marked as recommended.

**Background:** General Practice (GP) is the cornerstone of the NHS and has faced workload and workforce issues for many years. Enabling GP trainees to successfully complete their training and become independent practitioners is crucial to addressing these challenges. There is limited previous research exploring the postgraduate training experience specific to General Practice.

**Aim:** To explore GP Trainees’ experience of learning opportunities and support available during the three-year vocational training programme in the Southampton GP Education Unit.

**Design and Setting:** 15 semi structured interviews were undertaken December 2016-2018 with participants recruited from four cohorts. Telephone interviews lasting between 30-60 minutes were audio-recorded and transcribed verbatim; and analysed using inductive thematic analysis.

**Results and Conclusion:** Two themes were identified as affecting the training experience: ‘Developing clinical competence’ and ‘Sources of support’. Trainees perceived developing competencies relevant to General Practice was key. Busier hospital rotations with limited time for formal teaching were viewed less favourably. Trainers and peers were the main sources of support. Despite seeking a broad range of participants, interviewing trainees in difficulty was hard to achieve.

## Introduction

General Practice is at the centre of healthcare delivery in the United Kingdom and accounts for 90% of patient contact taking place in the National Health Service ( Royal College of General Practitioners, 2020). Since the formation of the NHS, General Practice continues to be the first point of contact and the gateway to accessing hospital and specialist care for most patients.

Over the past 72 years, the NHS has continually evolved and adapted to a changing social and political landscape (The Kings Fund, 2011). At present the NHS faces unprecedented challenges in the form of the COVID19 pandemic. Prior to the pandemic workload and workforce challenges in the form of rising patient demand, an ageing population, and funding and recruitment issues were key problems facing the NHS’ ability to meet the needs of patients. These challenges will likely continue beyond the pandemic. Successfully training GPs who can adapt to this changing context in which General Practice operates is crucial if we are to address these challenges and safeguard the vital role it plays in the structure of the NHS.

Not all GPs who begin training programmes successfully complete them. A gap in achievement exists between different demographic groups in training. This differential attainment is not unique to General Practice, it has been observed across all specialities at postgraduate level and also exists in undergraduate medical education and outside of medicine ( Woolf *et al.*, 2016). General Medical Council (GMC) data from the year 2018 on differential attainment shows pass rates varying across all specialities for post graduate examinations: For UK graduates 77.3% white graduates pass compared with 65.5% Black Minority Ethnic (BME) graduates; For International Medical Graduates (IMG) 45.7% white graduates pass compared with 44.3% of BME graduates ( GMC, 2020) ^b^. Studies have shown that understanding the learner’s experiences and how they interact with their learning environments can help understand and address some of these differences ( Mountford-Zimdars *et al.*, 2015).

There are limited studies exploring the training experience of GP trainees in depth. While there have been surveys and qualitative studies looking at the postgraduate training experiences, few studies are specific to General Practice. The General Medical Council’s National Training Survey has shown that General Practice is rated well by trainees with high overall satisfaction scores for the General Practice Programme across several rotations on the programme at a national level ( GMC, 2020) ^a^. A recent qualitative study looking at the influence of training experiences on career intentions of the future GP workforce found that trainees reported limited preparation for additional roles such as partnership within GP training, but the programme was still highly rated overall and most trainees felt they emerged from the programme with reasonable confidence in their clinical knowledge ( Spooner, Laverty and Checkland, 2019). The overall satisfaction scores for the General Practice component of the Programme in the Wessex Deanery between 2015-2019 was, on average, 91% ( GMC, 2020) ^a^. However, these data lack depth and richness of detail on learner’s experiences and context, which would be beneficial to refine and develop the support offered particularly for those trainees who face difficulties.

The aim of this qualitative study is to provide a deeper understanding of GP Trainees experience of learning opportunities available during their three-year vocational programme by identifying highlights and difficulties in training and the support mechanisms available.

## Methods

A qualitative research design using semi structured telephone interviews was conducted with GP trainees from Southampton’s GP Education Unit in the Wessex Deanery.

**Figure 1.**
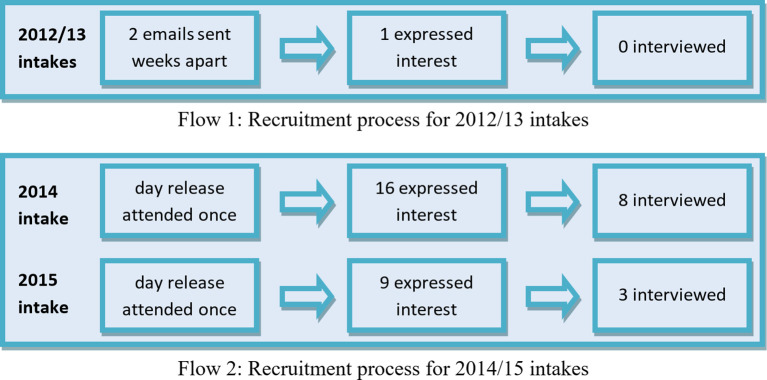
Schematic of the recruitment process

The interviews took place December 2016-2018 with trainees recruited from cohort years beginning 2012-2015. For the early part of the recruitment process, two emails were sent out to cohorts from 2012 and 2013 approximately 6 weeks apart. However, this recruitment approach yielded a poor response rate. As a result the research team decided to adopt a different approach and recruited participants by attending GP trainee educational meetings (Day Release) on two occasions, once to publicise the study to the 2014 cohort and a second time to publicise the study to the 2015 cohort (See Figure 1).

From the emails sent out to the 2012 and 2013 cohort, one person responded but did not respond to further emails so a telephone interview could not be arranged ( Figure 1). From the 2014 intake 16 trainees expressed interest in taking part and 8 returned their consent forms and were interviewed. Nine expressed interest from attending day release for the 2015 cohort, 3 returned consent forms and were interviewed. Four participants were recruited by word of mouth and snowball sampling.

Those wishing to participate were asked to register their interest by providing their names, email addresses and telephone numbers and were provided with a participant information sheet and consent form. They were advised they would be contacted upon receipt of the signed consent form. A semi structured topic interview guide was informed by the literature and developed collaboratively by the research team. The interview guide was divided into two sections. The first section focussed on the participants training experience with open ended questions asking them to describe each speciality year (e.g. Can you describe your ST1 year?). The second section explored support mechanisms and challenges with questions about highlights and difficulties in training and support mechanisms accessed. NFP the principal researcher and conducted all interviews by telephone and they lasted for 43 minutes on average (range 32-71minutes). The interviews were audio-recorded, anonymised and transcribed verbatim.

Interview data were imported into NVivo 11 and analysed inductively using thematic analysis ( Braun and Clarke, 2006). NFP led the analysis and developed initial codes. A qualitative researcher (CW) independently coded 25% of the interviews. The codes were discussed in a data meeting with the rest of the research team and a coding framework agreed. NFP coded the data using this framework and generated the final themes.

## Results/Analysis

A total of 15 interviews were conducted. One trainee out of the 15 self-identified as struggling on the programme. The mean age of participants was 31 years. 53% were White British and 60% male. 80% had attended state schools and 93% identified English as their first language and the same percentage had received their undergraduate medical degree in the United Kingdom. Table 1 presents the demographic and educational characteristics of the participants.

Two themes were identified as affecting the training experience which were ‘Developing Clinical Competence’ and ‘Sources of support’. There were negative and positive examples in each theme.

**Table 1.** Demographic and educational characteristics of participants.

Age	
Mean Range	**31** **29-40**
**Gender** Male Female	**9 (60%)** **6 (40%)**
**Ethnicity** White British South East Asian British Asian British Chinese Black British White Irish Mixed White and Chinese	**8 (53%)** **2 (27%)** **1 (7%)** **1 (7%)** **1 (7%)** **1 (7%)** **1 (7%)**
**First Language** English Pilipino	**14 (93%)** **1 (7%)**
**Primary Medical Qualification** United Kingdom Philippines	**14 (93%)** **1 (7%)**
**Secondary School** State Private	**12 (80%)** **3 (20%)**

**Developing Clinical Competence:** The importance of developing relevant clinical competence as a key learning need emerged through broad discussions exploring participants’ experience of their hospital rotations, their time in General Practice and assessments. When discussing hospital rotations, participants mostly valued rotations relevant to General Practice and the opportunities to learn and develop competencies that could be carried through to General Practice. This was perceived as more common in community-based rotations than acute hospital specialities. Palliative Care was an example of a community-based rotation that trainees felt had direct relevance to General Practice.

*‘...I guess palliative care is very relevant to general practice anyway. It’s always quite a challenge but I think it’s something that, in a lot of cases, general practitioners kind of take on as the lead, the key person in some cases. Obviously there is - you obviously try and involve the palliative team if it’s tricky case. But having that experience dealing with palliative patients in the secondary care setting as well has given me a bit more confidence with that, rather than referring always to the palliative care team which, although they can be very good, I know they can be quite bogged down - or not bogged down, but they are very busy anyway’ P8 Male*

Competence also emerged through participants discussing the increasing confidence they had developed as they neared the end of their training and consolidated their knowledge. Examples given were becoming more autonomous in their practice and relying less on their trainers. Passing exams, particularly the CSA, seemed to help this process with a few participants mentioning that they were able to relax in the period following CSA and enjoy General Practice.

*‘....my highlight is probably the last month. In the last month I really feel like my confidence is growing and I’m enjoying my job. And, yeah, I think the last month has probably been the highlight. It feels like things have kind of come together and I, yeah, like I said, I feel confident about managing patients. I know I still don’t know everything and I know there are loads of unknowns, but I’m actually enjoying the job.’ P6 Female*

Factors that hindered confidence varied between participants but included personal illness and significant events. However, these seemed transient and overcome with the support structures in place. A trainee who had taken time out of the programme due to illness described a phased return back to work and relying on their trainer more heavily for support in the initial months following their return. The trainee who self-identified as struggling on the programme said that exam failure had negatively impacted their confidence and was still in the process of trying to pass both the written and clinical skills examination.

Negative aspects in the theme of clinical competence included rotations in which trainees felt that there were limited opportunities for learning, long working hours and understaffed rotations. A few trainees mentioned the term ‘service provision’ in relation to their hospital experience-the perception that they were providing a service rather than developing the competencies required to be a GP.

*‘Because we were usually short-staffed SHO-wise and we had to do quite a lot of out of hours work. And there was - I can’t remember one teaching session we had on [rotation]. I can’t remember, I can’t remember one by any of the consultants or any of the seniors. It was all ad hoc. I learnt how to do lumbar punctures quite well when I was on there. And once they saw that I could do one lumbar puncture, they were like, “Right you do all the next ten lumbar punctures.” And it was kind of just like there was so much service to do rather than, you know, getting you to train and getting you to do audits and getting you to do projects. And even my educational supervisor didn’t even have a chance to really meet with me properly.’ P1 Male*

**Sources of Support:** A range of sources of support were identified by participants, but the GP Trainer and trainee peers were the key sources of support described. Other sources of support included programme directors, the wider practice staff and clinical supervisors on rotations. Trainers were valued for the accessibility and the day-to-day clinical support trainees had with them. The formality of the training relationship was described by some participants with a preference for a less formal training style.

*‘I suppose what was most useful as a supportive person was the GP trainer in the practice, I think. You’re in contact with them quite a lot, you see them daily, you can ask them for stuff during your clinics and I think they seemed to be the ones that were the most supportive certainly for your clinical side.’ P1 Male*

Peer support was another key source of support mentioned by trainees. Meeting up weekly with other trainees was valued by many of the participants as a safe place to discuss concerns, receive emotional support, form friendships and receive exam support, particularly for the Clinical Skills Examination. Trainees described getting together with their peers on an informal basis to prepare for the clinical skills examination. Forming these strong peer relationships seemed important to success on the programme.

“.... *a GP job can be very isolating, so meeting up weekly or however often we did, that was really nice to get together and just to talk through any concerns and talk through any difficult cases. That was actually really, really - I, I really enjoyed that. I thought that was the best support that the programme actually put in place for trainees.” P9 Female*

Participants also discussed support structures for specific circumstances such as the Professional Support Unit that helped one trainee take time out of training for illness. Participants also mentioned support provided in working less than full time.

The trainee we interviewed who struggled described difficulty finding consistent group of trainees with whom to revise for the CSA exam.

*“We have a group of ST3 .............there are five of us who practise, but the three were very good already so they didn’t practise with us anymore and they passed it first time. It’s just the two of us which was left who failed and we both are extended.” P14 Male*

Highlights of training mentioned by participants included ‘good rotations’ which were identified as rotations where there was protected time for teaching, supportive supervisors and learning opportunities. Developing confidence to practice independently in General Practice and exam success were also mentioned as highlights. Challenges included long working hours and staffing issues, keeping up with training requirements particularly the e-portfolio, exam preparation and negative clinical events.

*‘I think overall I would just say that I’ve really enjoyed it. And I think it’s been varied and stimulating and enjoyable. Yeah, I’ve met some really nice people. It’s been fun along the way. They organise some really good activity days and teaching days. And ST3 particularly I think has been excellent in terms of preparing for the CSA and the clinical teaching that we’ve had, I think it’s been very, very good. The only thing I would have preferred is to have more of that sort of clinical teaching in ST1. But overall it’s been very, very good.’ P3 Male*

## Discussion

This qualitative study exploring the GP training experience identified themes of ‘clinical competence’ and ‘sources of support’. Most trainees interviewed reported a positive training experience and felt the programme successfully prepared them to become independent practitioners. Participant’s descriptions of their training experience centred mainly on their hospital rotations, which were seen as opportunities to acquire knowledge and skills that might be relevant to General Practice. Busier rotations with limited time for formal teaching and rotations were deemed as less relevant to General Practice were viewed less favourably by trainees. Some of the busier rotations were viewed as service provision rotations.

There was a range of support systems mentioned by participants which included programme directors, trainers, peers, clinical supervisors and the wider practice team. The key sources of support were GP trainers and peer support. GP trainers were valued for the day to day clinical support within General Practice. The peer support system provided emotional support, friendship and exam support. Support for special circumstances in training were also mentioned by participants such as the Professional Support Unit to support through illness and time out of training as well as a communications skills group run for trainees with language difficulties.

Despite seeking a broad range of interviews, we were only able to interview one trainee in difficulty. This trainee identified many challenges that included multiple exam attempts, communication difficulties and finding peers to revise for the clinical skills examination. They had required an extension to their training.

## Conclusion

This study compliments and expands on the existing literature on GP training programmes. Studies have shown that General Practice is highly regarded by trainees for its one to one supervisory support and close clinical and exam support.This is also evidenced by the overall satisfaction scores for General Practice in the GMC National Training Survey ( Dale *et al.*, 2017; GMC, 2020) ^a^. Our results resonate with previous research which has shown that trainees view hospital-based speciality placements as variable, with some hospital-based specialities perceived as not always relevant to General Practice training and existing for the purpose of providing a service ( Dale *et al.*, 2017). This is likely to change with the upcoming reforms to the GP training Programme in which the time spent in General Practice will rise from 18 months to 24 months of the 3-year programme from 2022 ( British Medical Association, 2020).

Previous studies have shown an association between participants’ rating of how well their specialist training had prepared them for a career in General Practice and intended career plans for the first 3 years post qualification, with those who felt well prepared being more likely to become partners than those who felt less prepared ( Dale *et al.*, 2017). Studies have also shown that support and learning opportunities can impact on the appeal of a speciality that support, organised teaching, feedback and encouragement contribute to the appeal of the speciality ( Nicholson, Hastings and McKinley, 2016). Building the reputation of General Practice as a specialty supportive of its trainees is important for future recruitment and retention. Our research highlights that GP trainers and peer support are key to a positive training experience.

This qualitative study included participants with a broad range of demographics and provides a detailed exploration of the GP training experience, including learning opportunities and support available. It highlights key factors that trainees perceive as important and challenges to successfully completing a GP training programme.

The main limitation was problems in recruiting trainees who had experienced difficulty in training. This is despite multiple approaches of email invites, snowball sampling and attending day release. Thus the results may not be representative of their views. Further work is needed to understand why these trainees do not come forward, as it is important to be able to explore the challenges they face.

The study was conducted in one deanery, thus results may not be applicable to other regions.

## Take Home Messages

• Overrall, trainees felt the training programme successfully prepared them to become independent practioners and were positive about the training programme.
• A recommendation would be to review current hospital rotations to include as part of GP training to (wherever possible) focus on posts that are most relevant to General Practice competencies.
• Further research is required to explore why trainees in difficulty do not come forward, to gain a better understanding of the challenges they face.

## Notes On Contributors

**Nadia Fisher-Plum**, General Practitioner and Visitor, School of Primary Care and Population Sciences and Medical Education, University of Southampton, UK.

**Catherine Woods**, PhD, Research Fellow,School of Primary Care and Population Sciences and Medical Education, University of Southampton, UK.

**Johnny Lyon-Maris,** Honorary Professor and Associate Dean, Health Education England Wessex, GP Education Unit Southampton, UK.

**Sally Curtis,** PhD, Professorial Fellow Medical Education Faculty of Medicine, University of Southampton, UK.

**Geraldine M. Leydon**, PhD, Professor Healthcare Interaction, School of Primary Care and Population Sciences and Medical Education, University of Southampton, UK. ORCID ID: https://orcid.org/0000-0001-5986-3300

**Hazel Everitt**, Professor of Primary Care Research, School of Primary Care and Population Sciences and Medical Education, University of Southampton, UK. ORCID ID: https://orcid.org/0000-0001-7362-8403
