# Supplementary material for: GP Trainees experience of learning opportunities and support mechanisms on the GP training programme: a qualitative study
Source: MedEdPublish (2016). 2020 Dec 2;9:270. [Version 1] doi: 10.15694/mep.2020.000270.1 (PMC10697552; doi:10.15694/mep.2020.000270.1)
Supplement: Peer review history

# Reviewer report: Reviewer response for version 1

**DOI:** 10.21956/mep.20192.r31239

**Published:** 2021-02-23

**Authors:** Richard Hays

**Affiliations:** 1 James Cook University

This review has been migrated. The reviewer awarded 3 stars out of 5 This is a nice study that appears to have been done well, although I have a couple of questions that readers not familiar with the NHS and UK GP training might share. The sampling is not really clear. The table suggest that trainees from three different years were interview at the same time. Is that correct? If so, some would have completed training and be looking back, while others were new trainees looking forward. Both perspectives are of course interesting and worthy of exploration, but what actually happened? The thematic analysis also raised questions for me. Perhaps the questions constrained responses (the opening question does not seem to do this) but finding only 2 themes seems unusual. I would have thought that within 'developing competence' and 'sources of support' there might be sub-themes worthy of discussion. Appending the questions would help readers understand better what went into the analysis. A further limitation that should be mentioned is that the study involved participants in only 1 region. While many of the challenges are very similar elsewhere, interpretation is constrained, although this is an opportunity to go 'deeper' into the information to look at both local and national issues. Finally, the structure of the article could be improved. In qualitative analyses the results and discussion are often combined and that would make sense here. The Conclusion is longer than the discussion, whereas it should be just 1-2 paragraphs that summarise the key messages.

Reviewer Expertise:

NA

No decision status is available

# Reviewer report: Reviewer response for version 1

**DOI:** 10.21956/mep.20192.r31240

**Published:** 2021-02-18

**Authors:** Trevor Gibbs

**Affiliations:** 1 AMEE

This review has been migrated. The reviewer awarded 4 stars out of 5 As the authors point out, this does show similar results to previous research in the area. That does not preclude this paper however, since a point well made is best repeated- especially if little notice is made previously. It was interesting to see that those trainees in difficulty were reluctant to express their views. I would have liked to see the authors making more discussions and their thoughts regarding this but I am happy to recommend this paper to all those involved in VT training

Reviewer Expertise:

NA

No decision status is available
